# Cancer Mortality Pattern in Lagos University Teaching Hospital, Lagos, Nigeria

**DOI:** 10.1155/2015/842032

**Published:** 2015-01-05

**Authors:** Olakanmi Ralph Akinde, Adekoyejo Abiodun Phillips, Olubanji Ajibola Oguntunde, Olatunji Michael Afolayan

**Affiliations:** ^1^Department of Anatomic and Molecular Pathology, College of Medicine, University of Lagos/Lagos University Teaching Hospital, PMB 12003, Lagos, Nigeria; ^2^Department of Surgery, College of Medicine, University of Lagos/Lagos University Teaching Hospital, PMB 12003, Lagos, Nigeria

## Abstract

*Background.* Cancer is a leading cause of death worldwide and about 70% of all cancer deaths occurred in low- and middle-income countries. The cancer mortality pattern is quite different in Africa compared to other parts of the world. Extensive literature research showed little or no information about the overall deaths attributable to cancer in Nigeria. *Aims and Objectives.* This study aims at providing data on the patterns of cancer deaths in our center using the hospital and autopsy death registers. *Methodology.* Demographic, clinical data of patients who died of cancer were extracted from death registers in the wards and mortuary over a period of 14 years (2000–2013). *Results.* A total of 1436 (4.74%) cancer deaths out of 30287 deaths recorded during the period. The male to female ratio was 1 : 2.2 and the peak age of death was between 51 and 60 years. Overall, breast cancer was responsible for most of the deaths. *Conclusion.* The study shows that the cancers that accounted for majority of death occurred in organs that were accessible to screening procedures and not necessary for survival. We advise regular screening for precancerous lesions in these organs so as to reduce the mortality rate and burden of cancer.

## 1. Introduction

Cancer is a leading cause of death worldwide, accounting for 7.6 million deaths (about 13% of all deaths) in 2008, and is projected to continue rising, with an estimate of 13.1 million deaths in 2030 [1].

Worldwide, cancer deaths are more than the percentage of deaths caused by HIV/AIDS, tuberculosis, and malaria put together. It is the second leading cause of death in developed countries and is among the three leading causes of death for adults in developing countries [2].

According to the Office of National Statistic, UK [3], cancer related death accounted for an average of 156, 200 deaths per year between 2007 and 2009. The average cancer death per year for male over this period was 81,800, while the average cancer death per year for female was 74,400 during this period.

In the United State of America, an estimated number of 565,650 deaths occurred in 2008 as a result of cancer, representing 23% of total mortality that year [4].

In 2002, there were 6.7 million cancer deaths worldwide with less than 5% of these in sub-Saharan Africa but it has been estimated that, by 2020, cancer could kill 10.3 million people worldwide, with a 50 to 75 percentage increase in cancer mortality in sub-Saharan Africa [2].

About 70% of all cancer deaths in 2008 occurred in low- and middle-income countries.

Between 2000 and 2004 mortality due to cancer stood at 99,249 and 156,290 in Egypt and South Africa, respectively [5].

In a study that was carried out in Ghana by Wiredu and Armah between 1999 and 2000 [6], a total of 3659 cancer related deaths were recorded during the period.

From Ibadan cancer registry, the first such review in the same population reported that cancers of the reticuloendothelial system topped the list while the second review observed that liver cancer was commonest among males and cervical cancer was commonest among females [7–9].

In a retrospective study carried out in 2010 in the center of this study by Anorlu et al. [10], between 2002 and 2007, of the total of 2200 patients admitted into the gynecology ward, 104 deaths were recorded and 83 (88.3%) of these were attributable to cancer. Cervical cancer, though being preventable and potentially curable, was the leading cause of death. This is followed by ovarian and endometrial cancer. In a similar study carried out in Enugu by Anya et al. in 2006 [11], the leading cause of the 79 deaths out of the 2033 gynaecological admissions was cancer, mostly cervical, followed by choriocarcinoma, septic abortion, and ovarian cancer.

The cancer mortality pattern is quite different in Africa when compared to other parts of the world. This may be explained by the difference in the climate, diet, genetic factors, and so on. Cancers causing viral infections such as HBV/HCV, EBV, and HPV are responsible for up to 20% of cancer deaths in low- and middle-income countries [12].

There is lack or no efficient public policy on cancer issues across sub-Saharan African countries where infectious diseases like malaria and HIV/AIDS are the major public health concerns. In Nigeria, about 10,000 cancer deaths are recorded annually while 250,000 new cases are recorded yearly. The report of World Health Organization (WHO) reflects the alarming shortage of functional cancer control plans in sub-Saharan countries despite the enormity of socioeconomic havocs it poses to the countries. Among the 17% of African countries that have sufficiently funded cancer control program none are within sub-Sahara region. The data available on cancer mortality are rudimentary and grossly inadequate in Nigeria. Extensive literature research showed little or no information about the overall deaths attributable to cancer in Nigeria. Studies that encompass all cancers are rare. Most studies done on cancer mortality are based on specific cancers. Although, as informative as such studies are, they cannot sufficiently reveal the enormity of cancer havoc in Africa.

This study aims at providing data on the patterns of cancer deaths in our center using the hospital and autopsy death registers. It is the first of its kind in the past 20 years in our center.

## 2. Methodology

This is a 14-year retrospective study carried out in Lagos University Teaching Hospital from 2000 to 2013. Data were extracted from death registers in the wards and mortuary. These data include patient demographic data (age, sex, and tribe), clinical information, and histopathological type of the cancer. Patients with cancer who died from other causes were excluded. All patients who died of cancer irrespective of their age were included in the study.

The data were analyzed statistically using simple figures, ratio, percentages, table, and graphs.

## 3. Results

A total of 1436 cancer related deaths were recorded during the period of the study (Tables 1, 2, and 3) and the total number of deaths during this period was 30287 putting the percentage of death due to cancer as 4.74%. Overall, 5979 cases of cancers were diagnosed and managed in our facility during this period putting the overall cancer mortality rate at 24.0%. The yearly cancer mortality rate ranged between 11.9% and 42.1% (Table 4). The number of males that died of cancer during this period was 450 and that of females was 986 giving a male to female ratio of 1 : 2.2. The highest number of cancer deaths was recorded in 2011, and a total of 191 deaths were recorded. A bimodal age distribution pattern was noticed with peaks in the 0–10-year and 51–60-year age groups.

Overall, breast cancer was responsible for most of the deaths and accounted for 368 (28%) deaths during this period. This was followed by haematological malignancies which accounted for 129 (10%) deaths. Cervical, ovarian and liver malignancies were responsible for 125 (9%), 82 (6%) and 82 (6%) deaths, respectively.

All the 368 breast cancer deaths were female and the majority of these deaths occurred in young adult and the middle aged group, and these groups accounted for 294 (79.9%) deaths. 100 (27.3%), 93 (25.4%), 76 (20.7%), and 1 (0.2%) of the cases were seen in the age groups 41–50, 31–40, 21–30, and 11–20 years, respectively. Others were seen above the age of 50 years but no death was recorded above the age of 80 years. Hematological malignancies accounted for 129 deaths during this period. Of these, 30 (23%) deaths were seen in the 31–40 age group while 20 (15.5%) and 24 (18.6%) were seen within the age group 41–50 years and 0–20 years, respectively. The male to female ratio was 3 : 1. Of the 125 cases of cervical cancer 89.6% (112) were above 40 years of age. The 51–60 age group accounted for the highest prevalence with 35 (28%) deaths, which was closely followed by the 61–70 age group with 33 (26.4%) deaths. Twenty-four of the cases had metastasis as of the time of death. A total of 82 deaths were due to ovarian cancer which spread across all age groups except the 0–10 age group. The 41–50 age group accounted for the highest with 18 (22%) deaths and this was followed by 51–60 age group with 17 (20.7%) deaths. The >80 age group accounted for the least with 1 (1.2%) death. Three of the cases were bilateral and 10 had metastasis as of the time of death.

Prostate cancer was number seven on the list and accounted for the 64 deaths during this period. Most of these deaths occurred in the middle aged and elderly, and this group accounted for 94% (60). 15 (23%) deaths each were within the age groups 51–60 and 71–80 years during the period of our study. Of the 64 cases of death, 26 (40%) had advanced carcinoma as of the time of death and 80% of these advanced carcinomas had metastasized to other organs. Primary liver cell carcinoma accounted for 82 deaths during this period with most of the deaths occurring in the young adult and middle aged. 20 (24.4%) deaths each were recorded within the 31–40 and 51–60 age groups. This study shows that primary liver cell carcinoma does have a sex predilection; the male to female ratio is 1.1 to 1. However, death due to colorectal has a higher female occurrence with female to male ratio of 1.6 to 1. Ten of the cases had metastasized as of the time of death.

## 4. Discussion

This study showed that cancer death accounts for 4.7% of all deaths in Lagos University Teaching Hospital over the study period. This is almost equal to 5.6% cancer death in Korle Bu Teaching Hospital (KBTH) in 1996 [6]. The minimal difference could be due to variation in the duration of the studies. An increase of 1% per year was estimated in 2011 having 8.3% of increase in cancer mortality. The sharp reduction in cancer deaths in 2012 and 2013 was partly due to industrial unrest which caused loss of some working days. Our cancer death incidence is, however, much lower than the worldwide cancer mortality rate of 12% recorded by GLOBOCAN [1]. This low cancer mortality rate obtained in our study as against that of GLOBOCAN could result from the lower population of the individuals seen at the institution. Perhaps, a state wide or nationwide study might give a similar picture. Nevertheless, our report could be a good approximation of incidence of cancers for which mortality is high such as breast, haematological, and hepatocellular carcinoma and cancers with high survival rates such as prostatic cancers may be underrepresented in such series.

Reports based only on hospital autopsy series only, although having good diagnostic information, suffer from selective factors that operate in causing admission to a hospital and in having an autopsy. This may not be representative of deaths in a population. The published reports of the International Agency for Research on Cancer (IARC) by GLOBOCAN 2002 [2] estimates of cancer incidence, mortality, and prevalence for different sub-Saharan, where scanty death report and adequate cancer registry data do not exist, acknowledged the degree of detail and quality of the source of data. The report stated that both the accuracy of the recorded cause of death and the completeness of the registration vary considerably [2]. The estimates for country with little or no data could therefore be at best a mixture of real data, extrapolations from limited samples, and informed guesses. Reports based on extracts from autopsy and death registers like this series are expected to give the best contemporary information on the cancer mortality pattern.

There was bimodal incidence of cancer death in this study. The first modal incidence of cancer death was seen in childhood below the age of 10 years while the second was within the age range of 51–61 years. The high incidence of nephroblastoma and leukaemia could be responsible for the upsurge of death among children bellow the age of 10 years. This most likely contributes to the low life expectancy in the country. As reported in the studies from other sub-Saharan countries [8], the lower peak age of cancer death in adult as shown in this study and that of KBTH are a reflection of poor socioeconomic and inadequate health care of the people. Adult cancer deaths are seen mostly among elderly people in developed nations [10, 11].

This study showed an alarming female to male ratio of 2.2 : 1. The sex distribution is almost the same in other developed countries but this ratio is exceptional [6]. The reason for this female preponderance is probably due to the high prevalence and mortality of breast cancer among the females as well as the reduced prevalence of lung cancer in males and possibly high survival rate of prostatic cancer as most men with prostatic cancer usually die of other diseases. A much smaller ratio was recorded for sub-Saharan countries by GLOBOCAN [1]. Reports from developed countries showed almost equal or slightly increased M : F ratio as breast and cervical cancers are detected early and lung cancers take their toll in both sexes almost equally [10, 11].

From this study, in order of decreasing frequency, the 10 common organs involved in cancer death are breast, hematolymphoid, cervix, ovary, liver, colorectal, prostate, brain, kidney, and soft tissue. Ovarian and liver cancer tied for the 4th position.

This is almost similar to what was reported in Ghana with respect to the mortality due to breast, cervical, hematolymphoid, cervical, prostate, liver, and colorectal malignancies which are seen among the 10 common cases in both studies but with some slight differences in their relative positions [6]. However, while cancers of the kidney and soft tissue were relatively more common in our study, those of gall bladder, brain, pancreas, stomach, and lungs accounted for more deaths in Ghana study than ours. The reports from both African countries concur with those of GLOBOCAN 2002 [2] with regard to breast, cervix, hematolymphoid, colorectal, and prostate malignancies as the most common cause of cancer death. Kaposi sarcoma did not appear among the common cancer death in this study as opposed to the report of GLOBOCAN 2002. This could probably be due to the effect of its low mortality rate, better care, or a relative low prevalence of Kaposi among the HIV infected patients.

The commonest cause of cancer related death in our study is cancer of the breast, accounting for a total of 368 (26.4%) deaths during this period. All affected people were female with the majority of the deaths occurring in young adult and the middle aged group. No record of death due to breast cancer was seen in individuals above the age of 80 years in our study. Among the patients 12 (3%) were bilateral. The study in Ghana between 1999 and 2000 showed 17.24% of death due to breast cancer. Although, our study showed higher incidence than that seen in the Ghanaian study, the lower value obtained in Ghana is most probably due to shorter period of that study. GLOBOCAN indicated breast cancer as the second leading cause of death among women in Africa in 2008 [1].

Haematological malignancies and cervical cancer had the second and third position overall but among the females, cervical carcinoma ranked second. Cervical cancer is the most common cause of gynecological deaths. It ranked first in the study of Anorlu et al. in Lagos [10] and that of Anya et al. in Enugu [11].

It is shocking that the most common causes of cancer death among African women affect majorly two organs (breast and cervix) which are assessable to regular examination and screening, both of which are useful but not necessary for life and reproduction. The absolute low frequencies of breast cancer death in developed countries have been ascribed to early diagnosis and intervention. Therefore, it can be concluded that, putting aside any other factors of poor prognostic factors of breast cancer in Africa, the high mortality is due to inadequate public information, poor screening, and late presentation of our patients.

Of all the cancers occurring in sub-Saharan Africa, hematolymphoid malignancies have emerged as a major cause of morbidity and mortality accounting for 8.7% of incident cancer diagnoses and 9.9% of cancer deaths in the GLOBACAN 2008 report [1]. Non-Hodgkin lymphoma has been reported among common cancers in Sub-Sahara Africa [13, 14]. The high frequency of death by hematolymphoid malignancy in this study and that of Ghana is a reflection of the worse outcome in African patients with hematolymphoid malignancies where there is inadequate diagnostic and effective management of such cases.

Prostate cancer accounted for 5% of death in our study. This is lower than the 13% in a South African study [15]. Our study may not be a true reflection of the worldwide burden because it is institutional based, compared with cancer registry based analysis from South Africa. GLOBACAN 2008, however, clearly states an increasing trend in prostate cancer mortality in sub-Saharan Africa.

About 11.4% of cancer deaths were seen below the age of 20 years. In both sexes, haematopoietic, kidney, eye, and soft tissue were the topmost cause of cancer death in the paediatric age. This cancer mortality picture is comparable to the reported cancer morbidity trend in the Ghanaian paediatric population [16] and other developing African countries [17–29], with a preponderance of Burkitt's lymphomas, followed by retinoblastoma, nephroblastoma, and soft tissue sarcomas. However, low relative frequency of Burkitt lymphoma in our center compared with its higher frequency in other parts of Nigeria and other sub-Saharan African countries has been previously reported by Akinde et al. [30] and Tijani et al. [27]. The reason for this might be due to improved socioeconomic status with better control and treatment of malaria. There is also a low relative frequency of intracranial tumours in this study. This is in agreement with reports from other developing African countries but in contrast to the situation in Europe and North America and even in South Africa with their higher leukaemia and intracranial tumor frequency. However, a report of frequency rates of childhood cancers in Namibia, a developing African country, showed an intermediate or mixed picture with tumours of the central nervous system occurring most commonly (18%), followed by renal tumours (14%), leukaemia (12%), and lymphoma (11.5%) [31]. Also brain tumours occupied the second position in the paediatric malignancy mortality series, in Ghana [6]. The reasons for these differences require further investigation, though they might be due to over representation or absolute higher case frequency of brain tumours or higher case mortality rate in those countries.

## 5. Conclusion

This study shows that cancers constitute lesser cause of death in Nigeria comparable to findings in the literature. Female breast cancer, however, constitutes the majority of the deaths while prostate is largely responsible for cancer deaths among males.

An effective cancer screening management policy will prevent the majority of avoidable cancer deaths in Nigeria.

## Figures and Tables

**Table 1:** Table showing the sex and yearly distribution for cancer mortality.

Sex	2000	2001	2002	2003	2004	2005	2006	2007	2008	2009	2010	2011	2012	2013	Total	Percentage (%)
Male	22	15	16	29	17	19	27	27	40	63	50	53	28	44	450	31.3
Female	21	38	42	67	36	31	95	76	109	111	106	138	45	71	986	68.7

Total	43	53	58	96	53	50	122	103	149	174	156	191	73	115	1436	100

**Table 2:** Showing yearly incidence and age distribution.

Age/years	2000	2001	2002	2003	2004	2005	2006	2007	2008	2009	2010	2011	2012	2013	Total	Percentage (%)
0–10	7	8	4	11	3	6	17	11	6	10	14	2	7	5	110	7.7
11–20	3	3	1	7	2	1	7	2	1	5	13	4	1	4	54	3.8
21–30	3	6	5	4	7	4	3	9	12	5	9	7	2	4	80	5.6
31–40	6	7	11	15	10	10	21	22	33	27	22	24	7	25	240	16.7
41–50	6	4	12	19	8	8	29	8	25	41	33	48	15	17	273	19.0
51–60	9	13	10	21	6	12	23	23	32	40	25	43	16	29	304	21.1
61–70	7	7	10	12	6	7	15	17	27	30	26	33	16	18	231	16.1
>70	1	1	5	4	6	2	6	10	11	14	13	24	9	10	118	8.2
Unknown	1	4	1	3	1		1	1	2	2	1	6	0	3	26	1.8

Total	43	53	59	96	53	50	122	103	149	174	156	191	73	115	1436	100

**Table 3:** Showing organ specific and yearly distribution of cancer mortality.

Organs	2000	2001	2002	2003	2004	2005	2006	2007	2008	2009	2010	2011	2012	2013	Total	Percentage (%)
Breast	10	16	19	22	10	3	38	27	40	51	32	62	16	22	368	25.6
Haematology	6	8	1	10	4	8	14	12	7	19	19	7	5	9	129	9.0
Cervix		3	6	14	10	7	10	6	16	10	12	20	1	10	125	8.7
Kidney	0	5	2	2	3	2	5	2	3	6	5	3	1	1	40	2.8
Ovary	1	5	3	8	7	2	5	10	16	3	8	11	6	7	82	5.7
Soft tissue	3	1	2	2	3	0	2	4	2	5	7	0	2	5	38	2.6
Colorectal	3	1		1	3	2	4	6	9	10	5	14	7	11	76	5.3
Prostate	2		1	2	1	2	3	3	5	9	8	13	7	8	64	4.5
Liver	2	2	4	3	3	6	6	6	9	9	10	13	3	6	82	5.7
Lungs	2						5	1	6	7	1	7	2	6	37	2.6
CNS	2	1	0	3	0	5	6	3	12	10	9	4	1	0	56	3.9
Bone marrow	2	1			2	1	3	2	2		1	2	1	2	19	1.3
Stomach	4	1	2	0	1	1	1	0	4	2	1	3	2	1	23	1.6
Endometrium			2	2		1	3				3	3	3	3	20	1.4
Bladder	1		1	1	1	2	1		1	1	1	1	0	2	13	0.9
Eye			1	3	1		1	2	1		1		1	0	11	0.8
Others	5	9	14	23	4	9	15	19	26	32	32	28	15	22	253	17.6

Total	43	53	58	96	53	50	122	103	149	174	156	191	73	115	1436	100

**Table 4:** Showing the yearly cancer mortality rate.

Year	Cancer death	Cancer diagnosed	Cancer mortality rate (%)
2000	43	167	25.7
2001	53	364	14.6
2002	59	192	30.7
2003	96	228	42.1
2004	53	465	11.3
2005	50	204	24.5
2006	122	398	30.7
2007	103	419	24.6
2008	149	564	26.4
2009	174	545	31.9
2010	156	558	28.0
2011	191	625	30.6
2012	73	611	11.9
2013	115	639	18.0

Total	1436	5979	24.0
